# Habitat Preference and Spatiotemporal Habitat Use of the Endangered Flat‐Headed Cat in the Proposed Lundu Mangrove Protected Forest, Sarawak, Malaysia

**DOI:** 10.1002/ece3.74248

**Published:** 2026-10-14

**Authors:** Ahmad Ampeng, Hamden Mohammad, Happysupina Sait, Azahari Omar, Badrul Munir Md‐Zain, Shukor Md‐Nor, Carl Traeholt, Dayang Nuriza Abang Abdillah, Syafiani Osman, Jonny Ujeng, Rowina Ruth Cedric Jess, Abd Rahman Abg Anoi, Shannia June Ahmad, Roger Richard John Apiu, Bilhan Deli, Hazeri Yahya, Mohammad Saiful Mansor

**Affiliations:** ^1^ Conservation and Constitution Division (CCD), Forest Department Sarawak Kuching Malaysia; ^2^ Department of Biological Sciences and Biotechnology, Faculty of Science and Technology Universiti Kebangsaan Malaysia Bangi Malaysia; ^3^ Copenhagen Zoo Frederiksberg Denmark; ^4^ Faculty of Forestry and Environment Universiti Putra Malaysia Serdang Malaysia

**Keywords:** camera trap, conservation, endangered felid, habitat‐specific, nibong habitat

## Abstract

The flat‐headed cat *Prionailurus planiceps* is one of the least‐detected and most threatened felids in Southeast Asia, and limited field records have constrained understanding of its habitat associations and activity patterns. An initial biodiversity survey in the proposed Lundu Mangrove Protected Forest, Sarawak, yielded no detections; however, a subsequent photographic record in November 2021 prompted a targeted camera‐trap survey to document habitat‐specific detection patterns and spatiotemporal activity. Between August 2021 and May 2024, 148 camera trap units were deployed across multiple habitat types in this area. We obtained two still images and 57 video recordings, including documentation of an adult female with a kitten. Detection frequency varied markedly among habitat types, with 49 independent detections recorded in the nibong habitat, followed by nine in the buta‐buta habitat and only one in the nipah swamp. Individual cats were frequently recorded foraging in shallow water puddles within the nibong habitat, suggesting localised resource use that may influence detection rates. Temporal patterns of detection were highly variable, ranging from consecutive‐day occurrences to extended intervals of non‐detection spanning weeks or months, while several camera stations yielded only a single detection event throughout the study period. Activity was concentrated from the evening through late morning, with activity peaking around midnight, while detections remained evident during daylight hours, particularly in the nibong habitat, indicating temporal flexibility rather than strict nocturnality. These findings indicate that nibong habitat represents the most suitable habitat for the flat‐headed cat within the study area, while the buta‐buta habitat also provides important complementary habitat under intermediate hydrological conditions. This study provides important baseline ecological information to support future monitoring, habitat management, and conservation planning for the species in tropical regions.

## Graphical abstract

These findings identify nibong habitat as the primary habitat of the flat‐headed cat within the proposed Lundu Mangrove Protected Forest, while buta‐buta habitat also provides important complementary habitat under intermediate hydrological conditions. The results improve current understanding of the species' habitat use and temporal activity in tropical wetland ecosystems. This study provides important baseline ecological information to support future monitoring, protected area management, and conservation planning for the flat‐headed cat in Borneo.

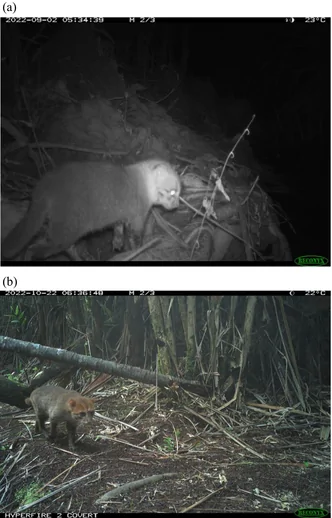

## 1 Introduction

The flat‐headed cat *Prionailurus planiceps* is among the rarest and most endangered small wild felids globally (Wilting et al. 2015), weighing only 1.5–2.5 kg (Muul and Lim 1970). Native to Southern Thailand, Peninsular Malaysia, Sumatra and Borneo (Sunquist and Sunquist 2009), it is highly adapted to a semi‐aquatic lifestyle, displaying morphological features such as a flattened head, semi‐webbed toes, and non‐retractable claws (Muul and Lim 1970; Leyhausen 1979). These adaptations make it an effective swimmer and specialised predator of slippery prey such as fish and amphibians. The species is strongly associated with lowland wetland ecosystems, including peat swamps, mangroves, and riverine forests, habitats that are increasingly threatened across its range (Wadey et al. 2017; Srisunthon and Chawchai 2020).

Despite its conservation status, little is known about its home range, population density, and behavioural ecology (Nowell and Jackson 1996; Bezuijen 2000). While typically described as nocturnal (Wilting et al. 2016), some evidence suggests crepuscular or diurnal activity patterns (Muul and Lim 1970; Davies and Payne 1982; Khan 1986; Payne et al. 1985; Traeholt and Idris 2011), that are often observed at riverine and wetland habitats where amphibians and fish are abundant (Muul and Lim 1970; Traeholt and Lim 1999; Malim and Yasuma 2000; Traeholt 2001). It has also been recorded to hunt rodents and birds (Payne et al. 1985; Khan 1986; Sunquist and Sunquist 2002), though Sunquist and Sunquist (2002) suggest that they only kill these without eating them. As a highly specialised predator of wetland ecosystems, the species is likely to play an important role in regulating populations of aquatic and semi‐aquatic prey, thereby contributing to trophic dynamics and the ecological functioning of freshwater and coastal wetlands (Wilting et al. 2016; Wadey et al. 2017). Owing to its close association with coastal wetlands, the species may also serve as an indicator of ecosystem integrity and environmental quality. However, knowledge of the species remains limited, highlighting the need for systematic field studies to better understand its habitat use and activity patterns. Improved knowledge of these aspects is essential for developing effective conservation and management strategies.

The IUCN Red List classifies the flat‐headed cat as endangered, primarily due to habitat loss and a lack of detailed ecological data (Wilting et al. 2015). In Sarawak, only a handful of camera trap detections have been published (Tisen and Azad 2013; Mohd‐Azlan and Thaqifah 2020), and no long‐term studies specifically targeting the species have been conducted. To date, no comprehensive biodiversity surveys or systematic ecological studies have been conducted within the proposed Lundu Mangrove Protected Forest (LMPF). This is likely due to the challenging accessibility of the area, which is dominated by extensive swamp forests and tidally influenced wetland habitats, making field surveys logistically demanding. In line with the Sarawak Government's initiative to expand the protected forest network to 6 million hectares, together with the recent confirmation of the endangered flat‐headed cat and other wildlife of conservation importance, the proposed LMPF is now being considered for gazettement as a Protected Forest. Given the conservation significance of the area and the limited ecological information available, this study investigated the habitat use, spatial occurrence, and temporal activity patterns of the flat‐headed cat across a mosaic of wetland habitats within the proposed LMPF. This study highlights the importance of establishing robust ecological baseline information to support evidence‐based conservation planning and long‐term management of this unique wetland ecosystem.

## 2 Materials and Methods

### 2.1 Study Site

This study was conducted in the proposed Lundu Mangrove Protected Forest (LMPF) (N 01°41′06.82″, E 109°55′16.21″), covering approximately 3582 ha in southwestern Sarawak, Malaysia (Figure 1). The area was first proposed for designation as a Protected Forest (PF) in 2000, and this proposed status has remained unchanged to the present day. The proposed LMPF is located far from human settlements and can only be accessed by approximately 45 min of travel by a 40‐hp boat from the nearest village. No agricultural activities or major developments have been recorded within the area. Fishing activities and the collection of snails, concentrated mainly along S. Chantong and S. Ban Bang, are carried out only occasionally by residents from nearby villages. The local communities primarily focus their fishing activities on the South China Sea rather than within the mangrove area.

**FIGURE 1:**
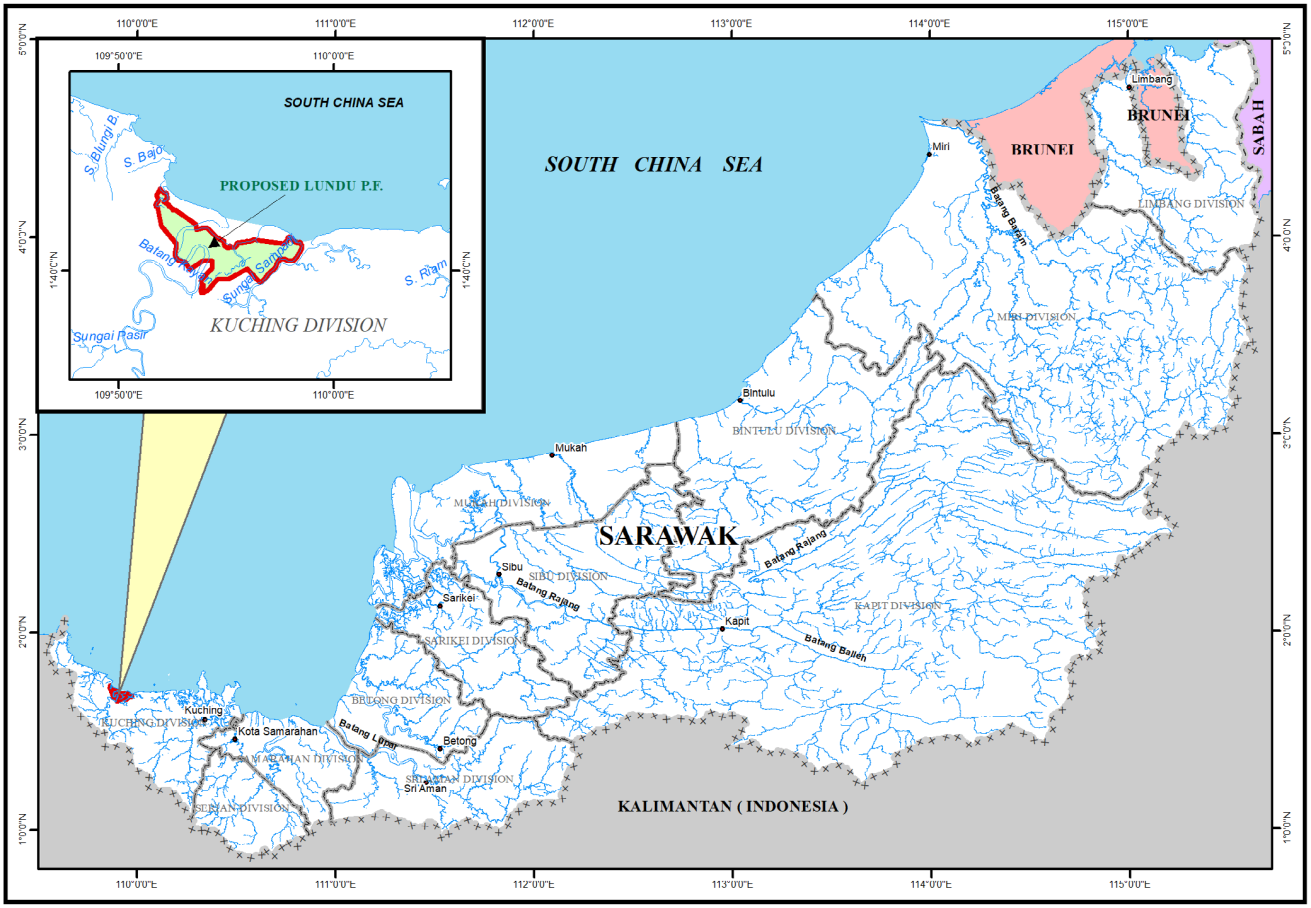
Location of the proposed Lundu Mangrove Protected Forest (LMPF) in Sarawak, Malaysian Borneo (inset).

The area ranges in elevation from near sea level (0–2 m asl) in tidal zones to approximately 15–25 m inland and elevated forest areas. The LMPF comprises a diverse mosaic of ten distinct habitat types (Figure 2), each exhibiting unique ecological characteristics and structural complexity. These forest types are irregularly distributed and spatially dispersed across the LMPF, contributing to a highly heterogeneous landscape. The study was conducted across two mangrove areas characterised by different dominant vegetation (nipah swamp and buta‐buta habitat), as well as nibong habitat (Figure 3). The mangrove habitat is dominated by *Rhizophora* spp. and *Sonneratia* spp., key species in intertidal coastal systems. The mixed mangrove habitat represents transitional zones where multiple mangrove species coexist. The nipah swamp habitat, dominated by *Nypa fruticans* , is typically found at low elevations (0–3 m asl) in brackish water environments, and is heavily influenced by tidal fluctuations. The buta‐buta habitat is dominated by *Excoecaria agallocha* , often found in slightly raised and less inundated zones. Both habitats are characterised by wet, muddy, and waterlogged substrates that support a diverse assemblage of coastal and estuarine flora and fauna. The nibong habitat, dominated by *Oncosperma tigillarium*, occurs in elevated areas (10–20 m asl) and, although not tidally influenced, contains freshwater tributaries that support amphibians and small fishes.

**FIGURE 2:**
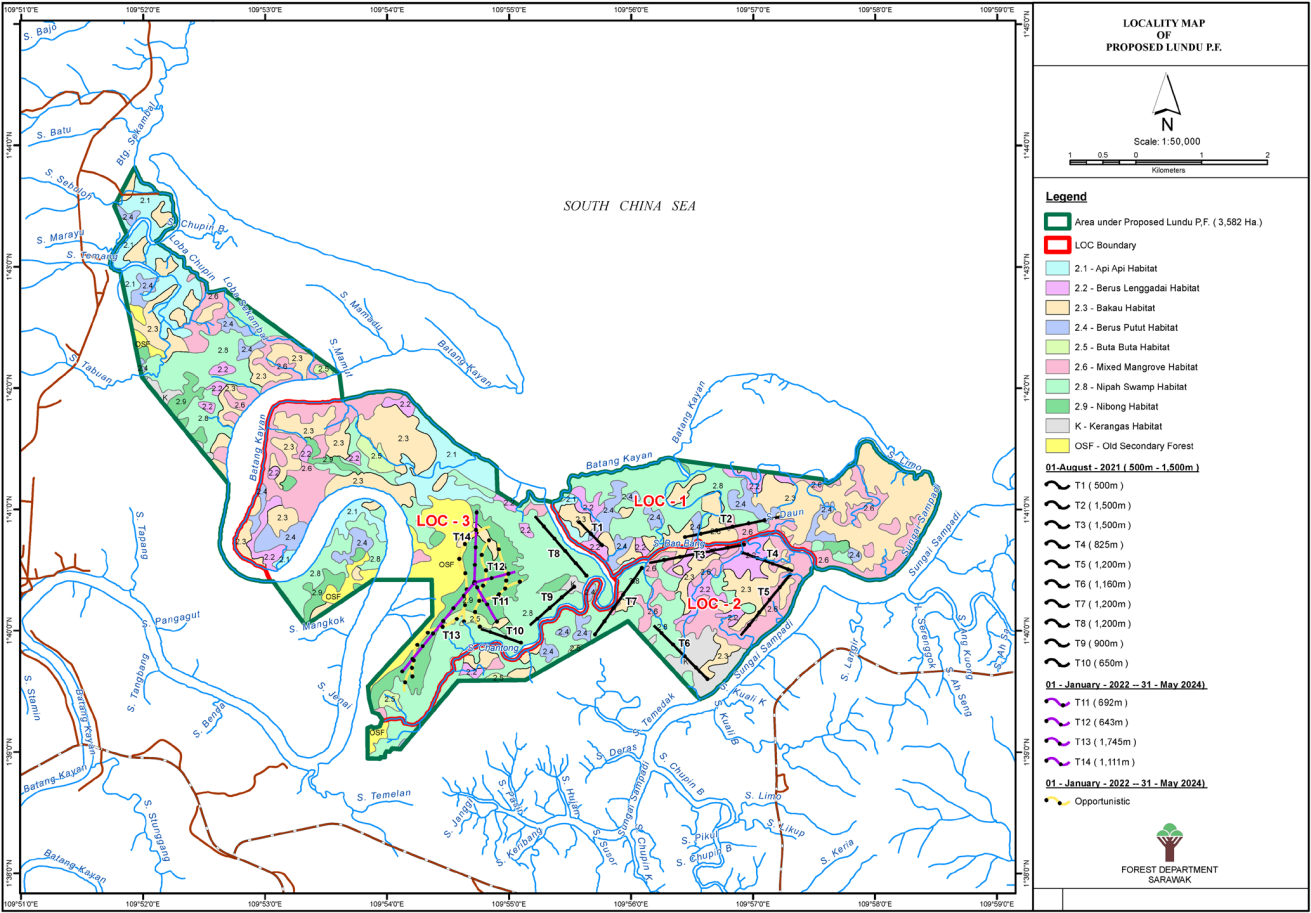
Distribution of camera‐trap transect lines across the selected habitat types within the proposed Lundu Mangrove Protected Forest. Source: Forest Department Sarawak.

**FIGURE 3:**
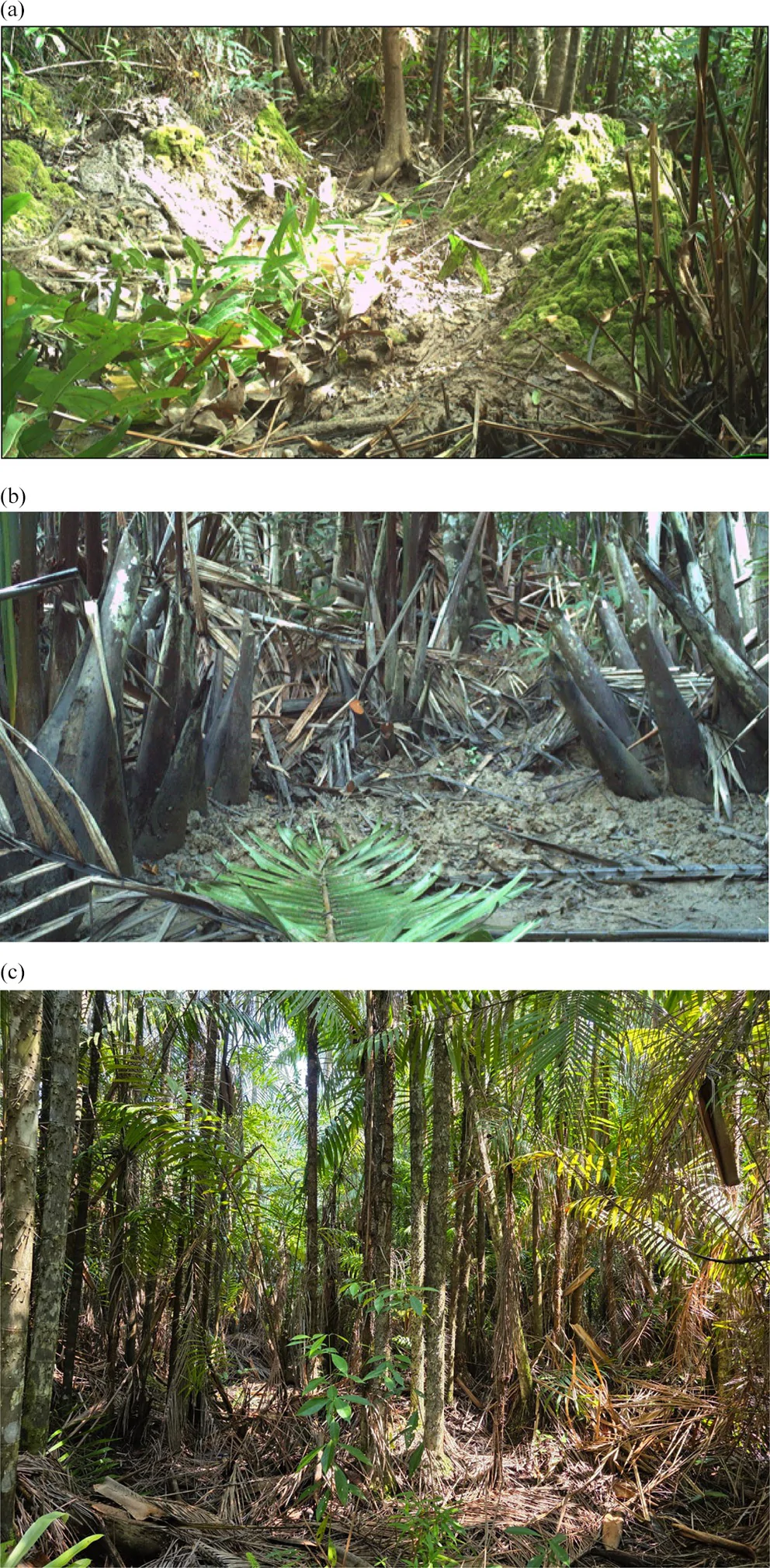
Habitat types within the proposed Lundu Mangrove Protected Forest: (a) buta‐buta habitat, (b) nipah swamp habitat and (c) nibong habitat.

### 2.2 Field Methods

The study area was divided into three major land sections, separated by two river systems (S. Chantong and S. Daun), and categorised into three sampling zones: Zone 1 (Loc‐1), Zone 2 (Loc‐2), and Zone 3 (Loc‐3). Loc‐1 and Loc‐2 are predominantly composed of mangrove and nipah swamp habitats at low elevations (0–3 m) and are both subject to tidal inundation. These zones are characterised by soft, muddy substrates and are dominated by *Avicennia* spp., *Rhizophora* spp., and *Nypa fruticans* . In contrast, Loc‐3 is situated at slightly higher elevations (up to ~20 m asl) and comprises nipah swamp, buta‐buta, and nibong habitats. Ten transect lines, ranging from 500 to 1500 m in length, were established approximately 300 m inland from the tidal boundary, running parallel to the riverbanks at all three survey locations. Following preliminary findings in November 2021 including confirmed records of the flat‐headed cat, four additional transects oriented perpendicular to the original transects were established exclusively at Loc‐3 to improve spatial coverage in areas with high detection rates.

A total of 148 camera trap units (Reconyx HyperFire II Covert, USA; Bestok, China; Best Guarder, China) were deployed across the study area from 1 August 2021, with camera placement stratified across the sampling zones according to habitat variability and field accessibility (Figure 2). Camera traps were installed systematically at 100 m intervals along transects established across the three study sites. In elevated dry areas that were not subjected to inundation, camera traps were mounted on small trees at approximately 2 ft. above ground level. In areas influenced by tidal inundation, camera traps were installed on small trees at heights of up to approximately 5 ft. above ground level, with the cameras angled downward toward the ground surface.

All cameras were programmed to capture three consecutive still images at 3‐s intervals, followed by a 10‐s video per trigger event, with no delay between triggers (Kelly et al. 2025; Harmange et al. 2025). Despite functioning as intended, the automatic transition time from still‐image mode to video recording on the camera units ranged from approximately 12 to 15 s per trigger event. This delay frequently resulted in incomplete footage of target animals, with recordings often capturing only partial anatomical features such as the posterior region or tail. To mitigate this limitation and enhance data quality, all camera units were reconfigured to operate exclusively in video recording mode effective 1 January 2022. Cameras were inspected approximately once per month to replace batteries, retrieve memory cards, and ensure that all units remained operational throughout the study period.

A single still image and video recording of flat‐headed cats were obtained at Loc‐3 after 113 days of camera trapping, on 21st November 2021, specifically at the ecotone between buta‐buta and nibong habitat, prompting methodological refinement. Camera deployment shifted from systematic transect‐based placement to a more targeted strategy. A total of 85 camera units at Loc‐3 were repositioned along three newly established transect lines within the nibong habitat, primarily near puddles and comparatively drier microhabitats. Additionally, six camera traps were deployed opportunistically outside the formal transect lines to enhance spatial coverage and improve detection probability. The remaining camera units were maintained at Loc‐1 and Loc‐2 to enable habitat‐based comparisons.

### 2.3 Data Analysis

Images captured by camera traps were sorted using DigiKam software (version 5.7.0–02). Species detections were defined as independent records (IR) when captures of a species at the same camera trap location were separated by more than 30‐min. The Encounter Rate (ER) was calculated as the number of independent detection events for a species divided by the total number of trap nights and multiplied by 100. The number of trap nights was calculated as the cumulative duration from camera trap deployment until memory card retrieval or the final image capture, accounting for any camera malfunctions or relocations. Images lacking wildlife, unidentifiable images, and human images were excluded from the analysis.

Habitat use by the flat‐headed cat was approximated using the naive occupancy estimate (ψ̂), calculated as the proportion of surveyed camera trap stations at which the species was detected at least once. This metric does not account for imperfect detection and therefore represents the observed proportion of occupied sites (MacKenzie 2005; Wintle et al. 2004). Habitat preference was evaluated using Jacobs' Index (Jacobs 1974), which compares the proportional use of each habitat type by the species (*r*) with its proportional availability in the study area (*p*). The index ranges from −1 to +1, where values approaching +1 indicate strong habitat preference, values approaching −1 indicate strong avoidance, and values near 0 indicate habitat use proportional to habitat availability. Jacobs' Index (*D*) was calculated as *D* = (*r*−*p*)/(*r* + *p*−2*rp*). Temporal activity patterns of the flat‐headed cat were analysed using Kernel Density Estimation (KDE) implemented in the R package overlap (Fleming and Calabrese 2017). Camera trap detection times were converted to decimal hours and subsequently transformed into radians for circular statistical analysis. Activity density curves were generated using non‐parametric kernel smoothing to estimate diel activity patterns over a 24‐h cycle.

### 2.4 Habitat Preference Analysis

Given the logistical challenges of tracking a cryptic species in extreme environments, and in the absence of telemetry data, habitat preference was inferred through camera trap visitation frequency per habitat type, following Mitterwallner et al. (2024).

## 3 Results

Camera trapping yielded 7426 independent records of wildlife species, with encounter rates varying substantially among habitat types. In all habitats, *Muridae* spp. recorded the highest encounter rate (2.908 in the buta‐buta habitat, 0.926 in the nipah swamp, and 3.544 in the nibong habitat), followed by treeshrews (*Tupaia* spp.; 2.125) and the small‐clawed otter ( *Aonyx cinereus* ; 2.116) in the buta‐buta habitat; the long‐tailed macaque ( *Macaca fascicularis* ; 0.258) and birds (*Aves* spp.; 0.112) in the nipah swamp; and treeshrews (*Tupaia* spp.; 0.720) and mousedeer (*Tragulus* spp.; 0.641) in the nibong habitat (Table 1). Based on independent records, flat‐headed cats (Figure 4) accounted for only 0.8% (59 detections; ER = 0.043) of all wildlife detections, including records of an adult female with a kitten. Most detections occurred in the nibong habitat (49 detections; ER = 0.056 encounter rates), followed by the buta‐buta habitat (9 detections; 0.087 encounter rates) and nipah swamp (1 detection; ER = 0.002 encounter rates).

**TABLE 1:** Independent records (IR) and encounter rates (ER) of mammal species recorded by camera traps across the three habitat types within the proposed Lundu Mangrove Protected Forest.

Species	Habitat						Total IR	Total ER
	Buta‐buta		Nipah swamp		Nibong			
	IR	ER	IR	ER	IR	ER		
Flat‐headed cat ( *Prionailurus planiceps* )	9	0.087	1	0.002	49	0.056	59	0.043
Bearded pig ( *Sus barbatus* )	75	0.725	5	0.012	53	0.060	133	0.096
Sunda pangolin ( *Manis javanica* )	3	0.029	2	0.005	3	0.003	8	0.006
Small clawed otter ( *Aonyx cinereus* )	219	2.116	9	0.022	16	0.018	244	0.176
Mouse deer (*Tragulus* spp.)	212	2.048	0	0.000	564	0.641	776	0.560
Small‐toothed palm civet ( *Arctogalidia trivirgata* )	7	0.068	0	0.000	19	0.022	26	0.019
Banded linsang ( *Prionodon linsang* )	0	0.000	0	0.000	1	0.001	1	0.001
Treeshrews (*Tupaia* spp.)	220	2.125	0	0.000	634	0.720	854	0.616
Squirrels (*Callosciurus* spp.)	23	0.222	0	0.000	226	0.257	249	0.180
Rats (*Muridae* spp.)	301	2.908	373	0.926	3119	3.544	3793	2.736
Mongoose (*Urva* spp.)	20	0.193	7	0.017	3	0.003	30	0.022
Malay weasel ( *Mustela nudipes* )	3	0.029	0	0.000	1	0.001	4	0.003
Proboscis monkey ( *Nasalis larvatus* )	27	0.261	0	0.000	54	0.061	81	0.058
Pig tailed macaque ( *Macaca nemestrina* )	0	0.000	0	0.000	2	0.002	2	0.001
Long‐tailed macaque ( *Macaca fascicularis* )	200	1.932	104	0.258	17	0.019	321	0.232
Silvered langur ( *Trachypithecus cristatus* )	0	0.000	3	0.007	0	0.000	3	0.002
Leopard cat ( *Prionailurus bengalensis* )	0	0.000	1	0.002	0	0.000	1	0.001
Birds (*Aves* spp.)	210	2.029	45	0.112	482	0.548	737	0.532
Monitor lizard ( *Varanus salvator* )	62	0.599	31	0.077	6	0.007	99	0.071
Crocodile ( *Crocodylus porosus* )	4	0.039	1	0.002	0	0.000	5	0.004

**FIGURE 4:**
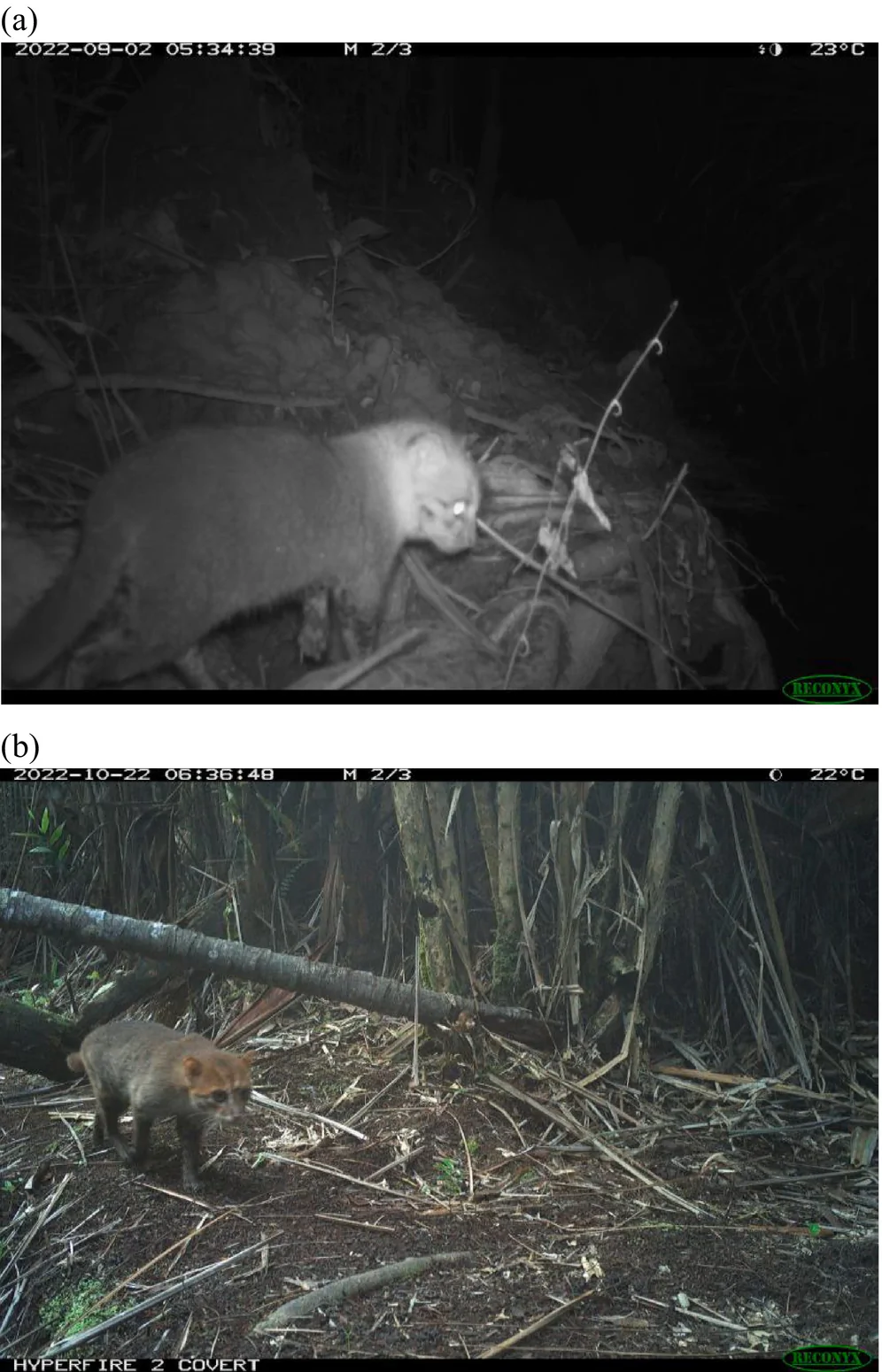
Camera‐trap photographs of the flat‐headed cat in the proposed Lundu Mangrove Protected Forest: (a) in the buta‐buta habitat and (b) in the nibong habitat.

Detection frequency varied spatially among camera stations. Most detections were concentrated at Loc‐3 within the nibong and buta‐buta habitats, while only a single detection was recorded at Loc‐2 in the nipah swamp and none in other habitats, including mangrove areas. Eighteen stations yielded only a single independent detection event throughout the study (Table S1). In the buta‐buta habitat, the cats were recorded on multiple occasions at two water puddle sites, with intervals between consecutive detections ranging from 2 days to 9 months. Within the nibong habitat, multiple stations recorded repeated independent detections, including consecutive‐day events (up to 3 days) as well as intervals spanning several weeks or months.

Habitat use by the flat‐headed cat differed significantly across habitats (*χ* ^2^ = 89.3, df = 2, *p* < 0.001). Bonferroni‐adjusted confidence intervals indicated that proportional use of nibong (0.712–0.944) was significantly greater than availability (0.333), confirming positive selection. In contrast, both buta‐buta (0.043–0.267) and nipah swamp (0.000–0.057) were used significantly less than expected. Jacobs' index further demonstrated strong selection for nibong habitat (*D*_*i*_ = 0.74), moderate avoidance of buta‐buta (*D*_*i*_ = −0.45), and near‐complete avoidance of nipah swamp (*D*_*i*_ = −0.95). In contrast, naïve occupancy was highest in the buta‐buta habitat (ψ = 0.364), followed by the nibong habitat (ψ = 0.231), whereas the nipah swamp exhibited very low occupancy (ψ = 0.022).

Temporal activity varied among habitats. In the nipah swamp (Loc‐2; *n* = 1) and buta‐buta habitat (Loc‐3; *n* = 9), detections were predominantly recorded during the night and early morning, with only one diurnal detection in the buta‐buta habitat. In contrast, detections in the nibong habitat (Loc‐3; *n* = 49) occurred throughout the diel cycle, with activity extending from nocturnal into diurnal periods and repeated daytime detections recorded throughout the study. The kernel density estimation (KDE) indicated that activity was concentrated from the evening through late morning, with activity peaking around midnight, while continued daytime activity in the nibong habitat suggests temporal flexibility rather than strict nocturnality (Figure 5).

**FIGURE 5:**
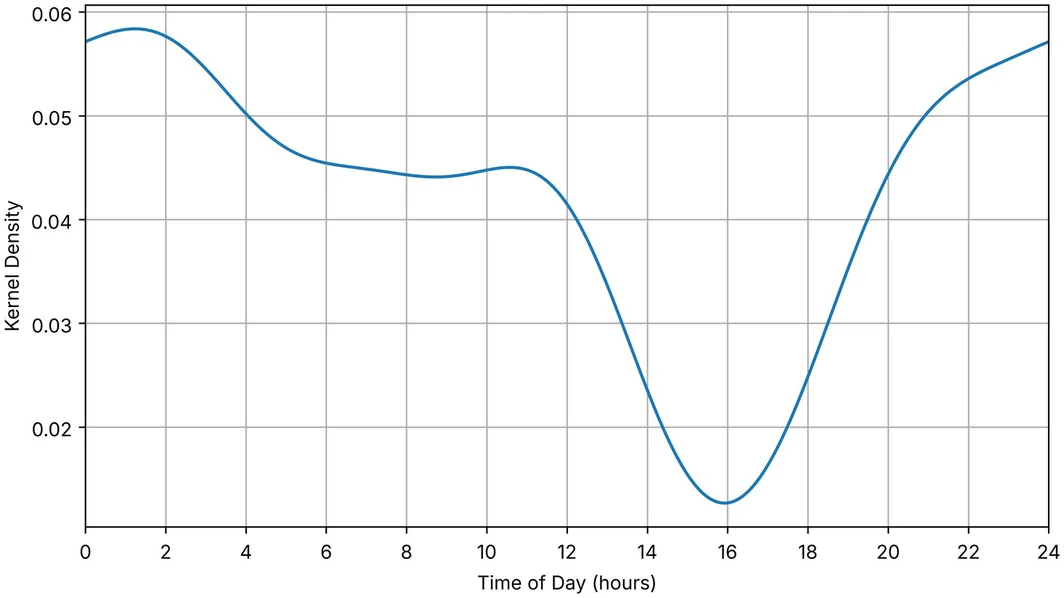
Diel activity pattern of the flat‐headed cat estimated using Kernel Density Estimation (KDE).

## 4 Discussion

The present study provides one of the first systematic insights into the occurrence and habitat use of the flat‐headed cat in Sarawak. Despite extensive camera trapping effort, the species represented only a small proportion of total wildlife detections (0.8%), highlighting its rarity and the difficulty of detecting this elusive felid. Such low detection frequencies are consistent with previous studies across its range, where the flat‐headed cat is often recorded infrequently even with extensive survey effort (Wilting et al. 2015). This pattern likely reflects both naturally low population densities and the species' specialised ecological requirements associated with wetland and riverine habitats (Wadey et al. 2017).

Habitat use analysis revealed a clear preference for nibong and buta‐buta habitats, with nibong showing significantly higher use compared to its availability in the landscape, which was further supported by consecutive‐day detections at several camera stations. In contrast, nipah swamp habitats were used significantly less than expected, indicating avoidance or limited suitability. Jacobs' index further supported this pattern, demonstrating strong positive selection for nibong habitat and strong avoidance of nipah swamp. The structural characteristics of nibong stands, which often occur along slightly elevated ground that is not inundated but remains close to water sources or puddles, may provide favourable hunting grounds where aquatic prey such as fish are accessible while still allowing efficient terrestrial movement (Wilting et al. 2015). Such habitat configuration may therefore represent optimal foraging environments for this semi‐aquatic felid.

In contrast, naive occupancy was highest in the buta‐buta habitat, indicating that a greater proportion of camera stations within this habitat recorded the species at least once. This pattern complements the habitat selection analysis, as naive occupancy reflects the proportion of occupied camera stations rather than the intensity of habitat use. The relatively higher occupancy in the buta‐buta habitat may be attributed to its intermediate hydrological conditions, being less frequently inundated than the nipah swamp while remaining closely associated with small water puddles and adjacent nibong stands. These habitat characteristics may provide suitable foraging opportunities while retaining relatively stable terrestrial substrates for movement. Along with this hydrological gradient, the frequently inundated conditions of the nipah swamp may reduce habitat suitability, whereas the elevated, non‐inundated nibong habitat appears to support more intensive and repeated use by the species, with the buta‐buta habitat representing an intermediate habitat that may function as a transitional foraging area.

Spatial patterns of detections further reinforced the importance of nibong habitat, particularly at Loc‐3, where most independent detections were recorded. Repeated detections at several camera stations suggest that this area may represent a frequently used activity zone within the study landscape. In buta‐buta habitat, detections were largely restricted to two water puddle sites, suggesting that localised microhabitat features such as small water bodies may influence habitat use. The long intervals between some detections, ranging from days to several months, may indicate irregular site visitation patterns or low individual density within the study area (Mohd‐Azlan and Thaqifah 2020).

Temporal activity patterns also varied among habitats. In buta‐buta and nipah swamp habitats, detections were largely restricted to nocturnal and early morning periods, which aligns with previous reports describing the flat‐headed cat as predominantly nocturnal (Danaisawadi et al. 2023). However, in the nibong habitat, the species was detected across both nocturnal and diurnal periods, with repeated daytime activity recorded. This broader activity pattern may reflect reduced disturbance levels or higher prey availability in this habitat, allowing individuals to forage throughout the day. Such flexibility in activity patterns has been occasionally reported in other small felids (e.g., Chen et al. 2016; Deng et al. 2022), particularly in habitats where prey availability or environmental conditions favour opportunistic hunting behaviour.

Overall, the results highlight the importance of nibong‐dominated wetland habitats for the flat‐headed cat within the study area. The strong habitat selection observed suggests that maintaining the structural and hydrological integrity of these habitats is critical for the persistence of the species. Although the relatively low number of detections and uneven spatial coverage limit inference on population size and fine‐scale habitat use, this study provides important baseline ecological information for the flat‐headed cat in Sarawak. These findings establish a foundation for future standardised and spatially replicated studies that incorporate models accounting for detection probability, while also emphasising the need to protect wetland‐associated habitats and maintain natural hydrological conditions as key components of effective conservation strategies for this highly specialised and threatened felid.

## 5 Implications for Conservation

The strong association of flat‐headed cat detections with nibong‐dominated habitats highlights the conservation importance of structurally complex wetland environments within mangrove landscapes in Borneo. These areas appear to provide a combination of accessible aquatic prey and relatively stable terrestrial substrates, which may facilitate both foraging and movement. Conservation planning in the proposed Lundu Mangrove Protected Forest should therefore prioritise the protection of nibong stands and adjacent wetland microhabitats, particularly areas containing small water bodies or puddles that may function as key foraging sites. The gazettement of this area as a protected forest would provide an important mechanism to safeguard these habitats and maintain the hydrological conditions necessary to support specialised wetland‐dependent species such as the flat‐headed cat.

Conservation measures targeting flat‐headed cats could simultaneously benefit numerous sympatric species dependent on peat swamp forests, mangroves, and estuarine ecosystems. More broadly, this study provides valuable baseline ecological information to support future conservation planning, long‐term monitoring programmes, species distribution modelling, protected area management, environmental impact assessments, and future reassessments by the International Union for Conservation of Nature (IUCN). The rarity of detections observed in this study highlights the vulnerability of the flat‐headed cat and the need for targeted monitoring and habitat protection across wetland ecosystems in Borneo. Given the ongoing pressures on coastal wetlands and mangrove systems from land conversion, hydrological modification, and human disturbance, proactive conservation measures are required to secure remaining suitable habitats. Long‐term monitoring using standardised camera trapping and spatially replicated surveys will be essential to better understand habitat use, activity patterns, and population status. Integrating such monitoring with landscape‐level wetland conservation strategies will be critical for ensuring the persistence of this highly specialised and threatened felid across its remaining range.

## Author Contributions

**Ahmad Ampeng:** conceptualization (equal), formal analysis (lead), funding acquisition (equal), investigation (lead), methodology (equal), project administration (equal), visualization (equal), writing – original draft (equal), writing – review and editing (equal). **Hamden Mohammad:** funding acquisition (supporting), project administration (supporting). **Happysupina Sait:** funding acquisition (supporting), project administration (supporting). **Azahari Omar:** funding acquisition (equal), project administration (equal). **Badrul Munir Md‐Zain:** supervision (supporting). **Shukor Md‐Nor:** methodology (equal), supervision (equal), validation (equal). **Carl Traeholt:** methodology (supporting). **Dayang Nuriza Abang Abdillah:** methodology (supporting), writing – review and editing (supporting). **Syafiani Osman:** investigation (supporting). **Jonny Ujeng:** investigation (supporting). **Rowina Ruth Cedric Jess:** investigation (supporting). **Abd Rahman Abg Anoi:** investigation (supporting). **Shannia June Ahmad:** formal analysis (supporting). **Roger Richard John Apiu:** investigation (supporting). **Bilhan Deli:** investigation (supporting). **Hazeri Yahya:** investigation (supporting). **Mohammad Saiful Mansor:** validation (equal), writing – review and editing (equal).

## Funding

This work was supported by the Sarawak State Government (P17‐B210) and the Federal Government of Malaysia (B23).

## Conflicts of Interest

The authors declare no conflicts of interest.

## Supporting information


**Data S1:** Camera trap detections of the Flat‐headed Cat (*Prionailurus planiceps*), with timestamps displayed and habitat types indicated.

**Table S1:** Month, date and time of flat‐headed cat presence recorded in three habitat types at Lundu Mangrove Protected Forest. Syst. = Camera trap set systematically on transects; Oppor. = Camera trap set opportunistically at puddles and along small streams.

## Data Availability

All the required data are uploaded as Data S1.
