# Supplementary material for: Habitat Preference and Spatiotemporal Habitat Use of the Endangered Flat‐Headed Cat in the Proposed Lundu Mangrove Protected Forest, Sarawak, Malaysia
Source: Ecol Evol. 2026 Oct 14;16(10):e74248. doi: 10.1002/ece3.74248 (PMC13662835; doi:10.1002/ece3.74248)
Supplement: Supplementary file 1 [file ECE3-16-e74248-s001.docx]

**Supporting Information**

Camera trap detections of the Flat-headed Cat (*Prionailurus planiceps*), with timestamps displayed and habitat types indicated.

**Nibong (Oncosperma tigillarium) habitat**

|  | **1.**  **Date:** 21 November 2021  **Time:** 0224 am  *Note: The very first image captured at the transition zone between the buta-buta (Excoecaria agallocha) habitat and the nibong habitat dominated by ***Oncosperma tigillarium*** triggered the establishment of additional perpendicular transect lines within the densely vegetated and physically challenging nibong habitat. This led to the subsequent detection of more flat-headed cat images within this habitat type. |
| --- | --- |
|  | **2. (Camera No. 97)**  **Date:** 31 January 2022  **Time:** 0852 AM |
|  | **3. (Camera No. 97)**  **Date:** 25 March 2022  **Time:**  0728 AM |
|  | **4. (Camera No. 97)**  **Date:** 31 March 2022  **Time:** 1152 AM |
|  | **5.**  **Date:** 05 April 2022  **Time:**  0851 AM |
|  | **6.**  **Date:** 16 April 2022  **Time:** 1224 PM  *** *Female with kitten, kitten not seen except in video*. |
|  | **7.**  **Date:** 13 May 2022  **Time:**  2344 PM |
|  | **8. (Camera No. 97)**  **Date:** 08 June 2022  **Time:** 0039 AM |
|  | **9. (Camera No. 97)**  **Date:** 01 July 2022  **Time:** 2212 PM |
|  | **10. (Camera No. 103)**  **Date:** 12 July 2022  **Time:** 1923 PM |

|  | **11. (Camera No. 103)**  **Date:** 13 July 2022  **Time:** 2127 PM | | | | |
| --- | --- | --- | --- | --- | --- |
|  | **12. (Camera No. 103)**  **Date:** 14 July 2022  **Time:** 1254 PM | | | | |
|  | **13. (Camera No. 103)**  **Date:** 1 August 2022  **Time:** 1253 PM | | | | |
|  | | **14.**  **Date:** 22 August 2022  **Time:**  1148 AM | | | |
|  | | **15.**  **Date:**04 September 2022  **Time:** 0305 AM | | | |
|  | | **16. (Camera No. 134)**  **Date:** 04 September 2022  **Time:** 0706 AM | | | |
|  | | **17.**  **Date:** 13 September 2022  **Time:** 0427 AM | | | |
|  | | **18. (Camera No. 134)**  **Date:** 16 September 2022  **Time:** 0332 AM | | | |
|  | | **19. (Camera No. 134)**  **Date:** 18 September 2022  **Time:**  2202 PM | | | |
|  | | **20. (Camera No. 134)**  **Date:** 21 September 2022  **Time:** 2045 PM | | | |
|  | | **21. (Camera No. 81)**  **Date:** 25 September 2022  **Time:** 1221 PM | | | |
|  | | **22. (Camera No. 81)**  **Date:** 03 October 2022  **Time:** 1107 AM | | | |
|  | | | **23.**  **Date:** 3 October 2022  **Time:** 1059 AM | |  |
|  | | | **24. (Camera no. 134)**  **Date:** 7 October 2022  **Time:** 2339 PM | |  |
|  | | | **25.**  **Date:** 11 October 2022  **Time:** 0122 AM | |  |
|  | | | **26.**  **Date:** 12 October 2022  **Time:**  0808 AM | |  |
|  | | | **27. (Camera No. 81)**  **Date:** 13 October 2022  **Time:**  0215 AM | |  |
|  | | | **28. (Camera No. 97)**  **Date:**13 October 2022  **Time:** 0227 AM | |  |
|  | | | **29. (Camera No. 97)**  **Date:** 22 October 2022  **Time:**  0647 AM | |  |
|  | | | **30. (Camera No. 81)**  **Date:** 22 October 2022  **Time:**  0236 AM | |  |
|  | | | **31. (Camera No. 134)**  **Date:** 25 October 2022  **Time:** 0600 AM | |  |
|  | | | | **32.**  **Date:** 29 October 2022  **Time:** 1103 AM |  |
|  | | | | **33. (Camera No. 81)**  **Date:** 07 November 2022  **Time:** 2231 AM |  |
|  | | | | **34.**  **Date:** 10 November 2022  **Time:**  0711 AM |  |
|  | | | | **35.**  **Date:** 16 November 2022  **Time:** 0201 AM |  |
|  | | | | **36.**  **Date:** 5 January 2023  **Time:** 2035 AM |  |
|  | | | | **37. (Camera No. 97)**  **Date:** 15 January 2023  **Date:** 1904 PM |  |

|  | **38.**  **Date:** 15 January 2023  **Time:** 1953 PM |
| --- | --- |
|  | **39.**  **Date:** 3 February 2023  **Time:** 0402 AM |
|  | **40.**  **Date:** 12 March 2023  **Time:** 1751 PM |

|  | **41.**  **Date:** 19 March 2023  **Time:** 0814 AM |
| --- | --- |
|  | **42. (Camera No. 97)**  **Date:** 17 March 2024  **Time:** 0602 AM |
|  | **43.**  **Date:** 17 March 2024  **Time:** 1031 AM |
|  | **44. (Camera No.97)**  **Date:** 19 April 2024  **Time:** 1939 PM |
|  | **45.**  **Date:** 26 April 2024  **Time:** 0122 AM |
|  | **46.**  **Date:** 1 May 2024  **Time:** 2327 PM |
|  | **47.**  **Date:** 2 May 2024  **Time:** 0019 AM |
|  | **48.**  **Date:** 12 May 2024  **Time:** 1230 PM |

**Buta-buta (Excoecaria agallocha) habitat**

|  | 1. **(Camera No.6)**   **Date:** 4 January 2022  **Time:** 0145 AM |
| --- | --- |
|  | **2.**  **Date:** 12 February 2022  **Time:** 1020 AM |
|  | **3. (Camera No. 1)**  **Date:** 23 Jun 2022  **Time:** 0549 AM |

|  | **4. (Camera No. 1)**  **Date:** 8 July 2022  **Time:** 2223 PM |
| --- | --- |
|  | **5.**  **Date:** 5 July 2022  **Time:** 1929 PM |
|  | **6. (Camera No. 1)**  **Date:** 13 August 2022  **Time:**  0237 AM |
|  | **7. (Camera No.6)**  **Date:** 1 September 2022  **Time:** 2020 PM |
|  | **8. (Camera No.6)**  **Date:** 2 September 2022  **Time:** 0534 AM |
|  | **9. (Camera No.6)**  **Date:** 5 October 2022  **Time:** 2357 PM |

**Nipah swamp (*Nypa fructican*) habitat**

|  | **1.**  **Date:** 22 April 2022  **Time:** 2122 PM |
| --- | --- |
