# Supplementary material for: Habitat Preference and Spatiotemporal Habitat Use of the Endangered Flat‐Headed Cat in the Proposed Lundu Mangrove Protected Forest, Sarawak, Malaysia
Source: Ecol Evol. 2026 Oct 14;16(10):e74248. doi: 10.1002/ece3.74248 (PMC13662835; doi:10.1002/ece3.74248)
Supplement: Supplementary file 2 [file ECE3-16-e74248-s002.docx]

**Table S1**: Month, date and time of flat-headed cat presence recorded in three habitat types at Lundu Mangrove Protected Forest. Syst. = Camera trap set systematically on transects; Oppor. = Camera trap set opportunistically at puddles and along small streams.

| No | **Nipah Swamp habitat** | | | | |
| --- | --- | --- | --- | --- | --- |
|  | **Month/Year** | **Date** | **Camera No** | **Time** | **No. of Individual** |
| 1 | April 2022 | 22 April 2022 | Syst. No. 3 | 21:22 | 1 |
| Total Camera Trap Stations | | | | | **46** |
| Flat Headed Cat Recorded at Systematic Transect | | | | | **1** |
| **TOTAL FLAT HEADED CAT RECORDED** | | | | | **1** |

| No | **Buta-buta habitat** | | | | |
| --- | --- | --- | --- | --- | --- |
|  | **Month/Year** | **Date** | **Camera No** | **Time** | **No. of Individual** |
| 1 | January 22 | 4 Jan 22 | Oppor. No. 6 | 01:45 | 1 |
| 2 | February 22 | 12 Feb 22 | Syst. No. 5 | 10:20 | 1 |
| 3 | June 22 | 23 Jun 22 | Oppor. No. 2 | 05:49 | 1 |
| 4 | July 22 | 5 Jul 22 | Syst. No. 3 | 19:29 | 1 |
| 5 | July 22 | 8 Jul 22 | Oppor. No. 2 | 22:23 | 1 |
| 6 | August 22 | 13 Aug 22 | Oppor. No. 2 | 02:37 | 1 |
| 7 | September 23 | 1 Sep 23 | Oppor. No. 6 | 20:19 | 1 |
| 8 | September 23 | 2 Sep 23 | Oppor. No. 6 | 05:34 | 1 |
| 9 | October 23 | 5 Oct 23 | Oppor. No. 6 | 23:57 | 1 |
| Total Camera Trap Stations | | | | | **11** |
| Camera Trap Deployed Systematically | | | | | **6** |
| Camera Trap Deployed Opportunistically | | | | | **5** |
| Flat Headed Cat Recorded at Systematic Transect | | | | | **2** |
| Flat Headed Cat Recorded Opportunistically at small puddles | | | | | **7** |
| **TOTAL FLAT HEADED CAT RECORDED** | | | | | **9** |

| **No** | **Nibong habitat** | | | | |
| --- | --- | --- | --- | --- | --- |
|  | **Month/Year** | **Date** | **Camera No** | **Time** | **No. of Individual** |
| 1 | Nov 21 | 21 Nov 2021 | Syst. No. 8 | 02:24 | 1 |
| 2 | Jan 22 | 31 Jan 2022 | Oppor. No. 19 | 08:52 | 1 |
| 3 | Mac 22 | 25 Mac 22 | Oppor. No. 19 | 07:28 | 1 |
| 4 | Mac 22 | 31 Mac 22 | Oppor. No. 19 | 11:52 | 1 |
| 5 | April 22 | 05 April 22 | Oppor. No. 24 | 08:51 | 1 |
| 6 | April 22 | 16 April 22 | Oppor. No. 31 | 12:24 | 2 |
| 7 | May 22 | 13 May 22 | Oppor. No. 8 | 23:44 | 1 |
| 8 | June 22 | 08 June 22 | Oppor. No. 19 | 00:39 | 1 |
| 9 | July 22 | 01 July 22 | Oppor. No. 19 | 22:12 | 1 |
| 10 | July 22 | 12 July 22 | Syst. No. 27 | 19:23 | 1 |
| 11 | July 22 | 13 July 22 | Syst. No. 27 | 21:37 | 1 |
| 12 | July 22 | 14 July 22 | Syst. No. 27 | 12:54 | 1 |
| 13 | August 22 | 01 Aug 22 | Syst. No. 27 | 12:53 | 1 |
| 14 | August 22 | 22 Aug 22 | Syst. No. 25 | 11:48 | 1 |
| 15 | September 22 | 04 Sep 22 | Syst. No. 13 | 03:05 | 1 |
| 16 | September 22 | 04 Sep 22 | Oppor. No. 41 | 07:06 | 1 |
| 17 | September 22 | 13 Sep 22 | Oppor. No. 48 | 04:27 | 1 |
| 18 | September 22 | 16 Sep 22 | Oppor. No. 41 | 03:32 | 1 |
| 19 | September 22 | 18 Sep 22 | Oppor. No. 41 | 22:02 | 1 |
| 20 | September 22 | 21 Sep 22 | Oppor. No. 41 | 20:45 | 1 |
| 21 | September 22 | 25 Sep 22 | Oppor. No. 21 | 12:21 | 1 |
| 22 | October 22 | 02 Oct 22 | Oppor. No. 21 | 11:07 | 1 |
| 23 | October 22 | 03 Oct 22 | Oppor. No. 28 | 10:59 | 1 |
| 24 | October 22 | 07 Oct 22 | Oppor. No. 41 | 23:39 | 1 |
| 25 | October 22 | 11 Oct 22 | Oppor. No. 45 | 01:22 | 1 |
| 26 | October 22 | 12 Oct 22 | Oppor. No. 45 | 08:08 | 1 |
| 27 | October 22 | 13 Oct 22 | Oppor. No. 21 | 02:15 | 1 |
| 28 | October 22 | 13 Oct 22 | Oppor. No. 19 | 02:27 | 1 |
| 29 | October 22 | 22 Oct 22 | Oppor. No. 19 | 06:47 | 1 |
| 30 | October 22 | 22 Oct 22 | Oppor. No. 21 | 02:36 | 1 |
| 31 | October 22 | 25 Oct-22 | Oppor. No. 41 | 06:00 | 1 |
| 32 | October 22 | 29 Oct 22 | Syst. No. 25 | 11:03 | 1 |
| 33 | November 22 | 07 Nov 22 | Oppor. No. 19 | 22:31 | 1 |
| 34 | November 22 | 10 Nov 22 | Oppor. No. 33 | 07:11 | 1 |
| 35 | November 22 | 16 Nov 22 | Oppor. No. 5 | 02:01 | 1 |
| 36 | January 23 | 05 Jan 23 | Oppor. No. 51 | 20:35 | 1 |
| 37 | January 23 | 15 Jan 23 | Oppor. No. 19 | 19:04 | 1 |
| 38 | January 23 | 15 Jan 23 | Syst. No. 11 | 19:53 | 1 |
| 39 | February 23 | 03 Feb 23 | Oppor. No. 55 | 04:02 | 1 |
| 40 | March 23 | 12 March 23 | Oppor. No. 61 | 17:51 | 1 |
| 41 | March 23 | 19 March 23 | Oppor. No. 53 | 08:14 | 1 |
| 42 | March 23 | 17 March 24 | Oppor. No. 19 | 06:02 | 1 |
| 43 | March 24 | 17 March 24 | Syst. No. 17 | 10:31 | 1 |
| 44 | March 24 | 19 March 24 | Oppor. No. 19 | 19:39 | 1 |
| 45 | April 24 | 26 April 24 | Oppor. No. 5 | 01:22 | 1 |
| 46 | May 24 | 01 May 24 | Oppor. No. 9 | 23:27 | 1 |
| 47 | May 24 | 02 May 24 | Oppor. No. 9 | 00:19 | 1 |
| 48 | May 24 | 12 May 24 | Oppor. No. 9 | 12:30 | 1 |
| Total Camera Trap Stations | | | | | **91** |
| Camera Trap Deployed Systematically | | | | | **27** |
| Camera Trap Deployed Opportunistically at small puddles | | | | | **64** |
| Flat Headed Cat Recorded at Systematic Transect | | | | | **10** |
| Flat Headed Cat Recorded Opportunistically at small puddles | | | | | **39** |
| **TOTAL FLAT HEADED CAT RECORDED** | | | | | **49** |
